# Treatment pathways and associated costs of metastatic colorectal cancer in Greece

**DOI:** 10.1186/s12962-022-00339-2

**Published:** 2022-02-14

**Authors:** Ioannis Sougklakos, Elias Athanasiadis, Ioannis Boukovinas, Michalis Karamouzis, Aggelos Koutras, Paulos Papakotoulas, Dimitra Latsou, Magda Hatzikou, Eugena Stamuli, Athanasios Balasopoulos, Aggelos Sideris

**Affiliations:** 1 Laboratory of Translation Oncology, University Hospital of Crete Medical School, Crete, Greece; 2 Ygeia Athens Hospital, Marousi, Greece; 3 Bioclinic Oncology Department Thessaloniki, Thessaloniki, Greece; 4 Molecular Oncology Unit, Department of Biological Chemistry, Medical School, National and Kapodistrian University of Athens, Athens, Greece; 5 University of Patras, Patras, Greece; 6 Theagenion Anticancer Hospital, Thessaloniki, Greece; 7 Pharmecons Easy Access, Athens, Greece; 8 Pierre Fabre Farmaka S.A., Agia Paraskevi, Greece

**Keywords:** Metastatic colorectal cancer, Braf600 mutation, Disease burden, Treatment pathways, Costs, Greece

## Abstract

**Objectives:**

Colorectal cancer (CRC) is the second leading cause of cancer in Europe, with 1.931.590 people newly diagnosed in 2020. The purpose of this study is the investigation of treatment options and healthcare resource metastatic CRC (mCRC) in Greece.

**Methods:**

This study is based on the information collected in November 2020 by an expert panel comprising of 6 medical oncologists from major public and private centers around Greece. A 3-round survey was undertaken, according to Delphi method. The treatment phases studied were: pre-progression; disease progression and terminal care. Pharmaceutical costs and resource utilization data were considered from the perspective of the Greek National Services Organization (EOPYY). RESULTS: Experts agreed that the anticipated prevalence of *RAS* mutation in mCRC is 47% (30% *RAS/BRAF* WT Left, 17% *RAS/BRAF* WT Right); 8% *BRAF* while, MSI-H/dMMR are found in 5% of mCRC tumors. Based on mutational status, 74.8% of patients receive biological targeted therapies in combination with fluoropyrimidine/based combination chemotherapy, as 1st line treatment, and 25.2% combination chemotherapy alone. At 2nd line, 58.6% of patients receive biological targeted therapies in combination with chemotherapy, 25.4% immunotherapy, 11% combination chemotherapy and 5% biological targeted therapies. At 3rd line 56% of patients receive combination chemotherapy, 28% biological targeted therapies, 10% biological targeted therapies in combination with chemotherapy and 6% immunotherapy. The weighted annual cost (pharmaceuticals and resource use cost) in 1st line per mCRC patient was calculated at €28,407, in 2nd line €33,568, in 3rd line €25,550. The annual cost beyond 3rd line per patient regardless mutation was €19,501 per mCRC patient.

**Conclusions:**

mCRC is a societal challenge for healthcare systems as the treatment is more prolonged but expand patients’ survival. Thus, reimbursement decisions should be based not just on the cost of the treatment, but on the magnitude of the benefit of its treatment on patients’ survival and quality of life.

**Supplementary Information:**

The online version contains supplementary material available at 10.1186/s12962-022-00339-2.

## Background

Colorectal cancer (CRC) is the third most frequent cancer in the world, accounting for 10% of new cancer cases in 2020 [1]. In addition, its global prevalence is expected to rise rapidly to > 3 million cases per year by 2040 [2]. In Europe, CRC is the second most frequent cancer. In 2020, CRC represented approximately 12.9% of new cancer cases in Europe [3]. It is estimated that by 2040, the number of incident cases of CRC in the four major European Union (EU) countries, which are Germany, United Kingdom, France, Italy will increase to 261,583 totally [4]. The expected increasing incidence rates of CRC is thought to be driven by various factors such as population ageing, dietary changes, increased diagnosis, and intensified survival [5]. In addition, the aforementioned factors, CRC is observed to occur more frequently in men (281,714 new cases) compared with women (238,106 new cases), yet it is not substantially different [1]. The slight disparities observed between CRC incidence in men and women is possibly influenced by the difference in behavioural and physiological patterns. Women tend to undergo screening more frequently, have a healthier lifestyle, and are more frequent users of hormone therapy which is associated with decreased risk of CRC [6].

In Greece there were 6529 new cases of CRC in 2020 (3rd most common cancer, accounting for 10.1% of all cancers apart from non-melanoma skin cancers) and 3431 deaths (10.3% of the total number of cancer deaths, 2nd most common cause of cancer related deaths) [1]. The 5-year prevalence of CRC was estimated at 18,545 patients [1].

The colon is affected twice as often as the rectum in most cases of CRC [7]. The majority of CRC cases are spontaneous and arise from benign neoplasms called polyps that can eventually evolve into cancer over time via a multistep mechanism. Several risk factors are associated with the occurrence of CRC, including age and hereditary factors [8]. A substantial number of environmental and lifestyle risk factors also play an important role in the development and progression of CRC. Such factors include obesity, physical inactivity, alcohol and/or tobacco consumption and high consumption of red meat and saturated fat [8].

Furthermore, metastatic colorectal cancer (mCRC) arises from a precursor lesion, the adenomatous polyp, which forms, as a result of epithelial cell hyperproliferation and crypt dysplasia. Progression from this precursor lesion to CRC is a multistep process, involving also alteration in suppressor genes and cell regulation abnormalities, and has a natural history of 10–15 years [9]. Hyperactivation of *RAS* or *BRAF* of the MAPK signalling cascade is the most common oncogenic event in CRC tumorigenesis; it can be hyperactivated via several mechanisms, including mutations [10].

Diagnosing CRC at its earliest stage can improve patient prognosis, as tumours grow and tend to metastasize with time [11]. However, CRC diagnosis is often delayed as it is usually asymptomatic at a very early stage of the disease and its earliest symptoms are not disease-specific but common to other gastrointestinal disorders. The diagnostic tools used to identify CRC in patients with associated conditions include: colonoscopy, biopsy, computed tomography (CT)/Positron Emission tomography (PET) scan. Biomarker testing is also used for diagnosis and classification purposes of CRC. The most commonly used and appropriate biomarkers in patients with mCRC are: *RAS* (*NRAS* and *KRAS*), *BRAF* and MSI [12]. The mutation status is a crucial criterion to be considered in CRC management as some biomarkers have a diagnostic, prognostic and/or predictive value. It is estimated that approximately 50% of patients will develop metastases throughout the disease course and 25% of CRC patients are diagnosed at the metastatic stage [13].

The cost of cancer care has emerged as a major concern for the healthcare system, patients and caregivers. The total annual cost of mCRC for the EU27 countries from a societal perspective was estimated to be €13.1 billion, accounting for 10% of total cancer costs in the EU in 2009 [14]. The breakdown of which was 42% direct costs (€5.57 billion), 36% productivity loss costs (€4.69 billion) and 22% informal care costs (€2.84 billion). Additionally, the total annual cost of mCRC in France, Germany, Italy and the UK in 2015 were estimated to range from €1667 million to €4171 million [15].

To the best of our knowledge, there is lack of literature that estimates the direct cost of metastatic colorectal cancer in Greece, although CRC was the second most costly cancer in terms of direct costs in the EU27 countries [14]. Thus, the aim of this study was to map the treatment pathway in metastatic colorectal cancer in Greece and to investigate the health care resource use associated with the management of the disease.

## Methods

The methodology followed was based on a two-step approach. First, the local treatment pathways and associated resource use were identified emerging from a panel of experts. Secondly, the total cost for each pathway was estimated, by assigning unit costs to resource use items.

### Local treatment pathway and resource use

An expert panel of 6 medical oncologists of public and private sector with expertise in metastatic CRC was convened, in order to map the current local treatment algorithm and associated health care resource use. The patient pool represented by the physicians has been estimated at 750 patients annually, regardless of the type of metastasis.

For the purposes of data collection, a questionnaire was developed, regarding the therapeutic and pharmaceutical algorithm followed in Greece for all types of mCRC, followed by resource use consumption at all stages. The treatment phases studied were: pre-progression; disease progression and terminal care. After progression it was assumed that the patient changes treatment line. The questionnaire was structured, on the basis of a comprehensive literature search of published studies on treatment patterns and clinical guidelines at a local and global level [12]. The first draft of questionnaire was reviewed and validated by an expert clinician. Comments were incorporated, and final version of the questionnaire was distributed and answered by all experts (Additional file 1).

Data collection was performed during an expert panel and the data elicitation method was the Delphi technique, which aims at consensus-building [16]. This method uses multiple iterations and a feedback process that allows and encourages the selected Delphi participants to reassess their initial judgments about the information provided in previous iterations in light of other participants’ input. A three-round Delphi approach was used. Firstly, the validated questionnaire was answered by all experts. Secondly the results displaying mCRC treatment algorithm and resource use were provided to expert panel, asking to confirm their original responses and/or answer other questions based on group feedback from the first round. In the third round, the oncologists met for a consensus meeting.

### Total costs for each pathway

Unit costs were retrieved from publicly available sources, ministerial gazette and other healthcare sources. The perspective adopted was that of the Greek National Services Organization (EOPYY), thus all cost estimates reflect the economic burden of the disease to EOPYY.

Activity based costing method was followed for the estimation of costs. Only direct medical costs were considered, which consisted of oncology drug costs, costs of resource utilization associated with medical consultations, home care, hospital visits, laboratory tests, imaging examinations and procedures. The cost analysis in this study is presented on monthly and annual basis.

The estimation of the weighted cost per patient per mutation per line was based on percentages of each pharmacological treatment reported by the expert panel by adding the additional cost of resource utilization. Similar disease management/resource consumption was considered for all mCRC patients independent of mutation. According to expert panel the cost of progression was allocated as follows: 1st line—1 relapse, 2nd line-average of 1.25 relapses, 3rd line—average of 1.75 relapses.

To estimate pharmaceutical costs, the average price per mg was calculated based on the hospital prices per package, for all packages marketed in Greece. Prices were taken from the latest Price Bulletin [17]. In Table 1 are presented the pharmaceutical costs. Also, the cost of vial administration as 8-h day care was 40€ [18]. PORT-A-CATH (€113) was added in the cost of chemotherapies [19]. It is important to mention that in the analysis the Greek hospital prices per package were calculated including 5% mandatory discount (EOPYY reimbursement price) [20]. To take into account the dosage changes for the regimens caused by adverse events, relative dose intensity (RDI) was used in all treatments. Table 1 shows the hospital prices without taking into consideration any potential discounts i.e., Rebate and clawback or negotiated prices between pharmaceutical companies and negotiation committee, as this information is confidential.

**Table 1:** Pharmaceutical costs

	Administration	Strength/pack	Hospital prices—5%, unit cost (€)	Cost per mg (€)
Irinotecan^a^	IV	20 mg/ml × 15 ml	147.31	0.47
Folinic acid^a^	IV	10 mg/ml × 20 ml	11.2	0.03
Fluorouracil^a^	IV	50 mg/ml × 100 ml	14.14	0.0028
Cetuximab	IV	5 mg/ml × 20 ml	132.26	1.32
Panitumumab	IV	20 mg/ml × 20 ml	1100.28	2.75
Bevacizumab	IV	25 mg/ml × 4ml	204.52	2.05
Aflibercept	Intravitreal	25 mg/ml × 4 ml	250.27	2.50
Oxaliplatin	IV	5 mg/ml × 10 ml	19.82	0.40
Capecitabine	Oral	150 mg/tab × 60 tabs	10.66	0.002
Nivolumab	IV	10 mg/ml × 24 ml	2106.87	8.78
Pembrolizumab	IV	25 mg/ml × 4 ml	2229.89	22.30
Encorafenib	Oral	75 mg/ml × 42 caps	1046.92	0.33
Tas102 (Trifluridine-tiporacil)	Oral	(15 + 6.14) mg/tab × 20 tabs	445.92	1.05
Regorafenib	Oral	40 mg/tab × 84 tabs	1884.71	0.56
Mitomycin-C	IV	2 mg/vial × 10 vials	15.91	0.84

For dosing schedules dependent on body weight or surface area, an average body weight of 70.7 kg and body surface area of 1.79 m^2^ were used

^a^In the cases of Irinotecan, Folinic acid and Fluorouracil the cost per mg reflects the average price of generics/off patent which was used in the analysis

Costs associated with resource utilization were retrieved from EOPYY Health benefits list [19] and are presented in Table 2.

**Table 2:** Unit costs of resource utilization

	Unit costs (€)
**Medical consultations**	
Medical oncologist consultation	10.80
Radiation oncologist consultation	10.80
Oncology nurse visit	5.00
GP consultation	10.00
Psychology specialist consultation	15.00
Surgeon consultation	10.00
Dermatologist consultation	10.00
**Hospital visits**	
Inpatient stay (oncology/general ward)	80.00
Emergency department visit	No cost
Day hospital visit	40.00
**Examinations**	
Whole-body CT	140.00
Brain MRI	236.95
Brain CT-scan	71.11
Chest radiograph	4.05
PET-CT scan	700
Bone scan	34.42
Blood test (CBC, CMP)	2.88
**Procedures**	
Radiotherapy fraction	250.00
Surgical intervention	1306.00
Home care	
BSC physician/nurse visit	7.30
Home aid (non-medical specialist) visit	5.00

## Results

The mean age of mCRC patient reported by the expert panel is 64.6 years, with 1.79 [Standard Deviation (S.D). 0.23] body surface area (BSA) and 47.3% male. Among mCRC patients, 36.6% will become metastatic, from whom *RAS* mutation occurs in 45% of patients, *BRAF* mutation occurs in 8%, *RAS/BRAF* WT left occurs in 30% and *RAS/BRAF* WT right occurs in 17%. MSI-H dMMR occurs in 5% of patient in disease progression (2nd line).

Table 3 presents the pharmaceutical treatment options at 1st, 2nd and 3rd line. More specific, among RAS mutated patients, at 1st line 35% receive combination of Folfox and Bevacizumab, at 2nd line 20% receive combination of Folfiri and Bevacizumab and at 3rd line 50% receive Tas102. Regarding BRAF mutated patients at 1st line, 30% of them receive combination of Folfoxiri and Bevacizumab, at 2nd line 25% of them receive combination of Folfiri and Bevacizumab or combination of Encorafenib and Cetuximab, and at 3rd line 50% of them receive combination of Encorafenib and Cetuximab. Regarding *RAS/BRAF* WT left patients at 1st line, 40% of them receive combination of Folfiri or Folfox and Panitumumab, at 2nd line 30% of them receive Folfiri and Bevacizumab and at 3rd line 40% of them receive Tas102. For RAS/BRAF WT right patients, at 1st line, 30% of them receive combination of Folfoxiri and Bevacizumab, at 2nd line, 25% of them receive combination of Folfiri and Panitumumab and at 3rd line, 40% of them of them receive Tas102. Finally, 100% of MSI-H dMMR patients receive immunotherapies (95% pembrolizumab and 5% combination Nivolumab and Ipilimumab) at 2nd line and 70% Folfiri at 3rd line. After third line, the disease management and pharmaceutical treatment continues on the best alternative treatment basis, in which the majority of patients (65%) receive Tas102, 25% regorafenib and 10% 5FU/MITO-C. 25% regorafenib and 10% 5FU/MITO-C.

**Table 3:** Treatment options per mutation per line (as % of patients)

	RAS mutated			BRAF mutated			RAS/BRAF WT left			RAS/BRAF WT right			MSI-H dMMR	
	1st line	2nd line	3rd line	1st line	2nd line	3rd line	1st line	2nd line	3rd line	1st line	2nd line	3rd line	2nd line	3rd line
Folfiri	5	10	10	10	20				20	5				70
Folfiri & Cetuximab							15	5	10		15	10		
Folfiri & Panitumumab							20	5			25	10		
Folfiri & Bevacizumab	15	20		15	25		10	30		20	10			
Folfiri & Aflibercept		15			10			10			10			
Folfox	5	5	5	10							10			
Folfox & Cetuximab					10		15	5						
Folfox & Panitumumab							20	5						
Folfox & Bevacizumab	35	15		5			10	28		10	10			
Folfoxiri	3			20						20				
Folfoxiri & Panitumumab										5				
Folfoxiri & Bevacizumab	12			30						30				
Capecitabine			5											
Capecitabine and Oxaliplatin (CapOX)	3	10												
CapOX & Bevacizumab	12	10		10										
Capecitabine & Bevacizumab	10	10					10	10	10	10	10	10		
Tas102			50			40			40			40		
Regorafenib			30			10			10			10		
Encorafenib & Cetuximab					25	50								
Cetuximab									10			10		
Panitumumab												10		
Immunotherapies		5			10			2			10		100	30
Total	100	100	100	100	100	100	100	100	100	100	100	100	100	100

Regarding resource utilization, the periods pre- progression were reported on a monthly basis and were considered of similar disease management as presented in Table 4.

**Table 4:** Resource utilization during stable disease (pre progression), at disease progression and beyond third line

	Pre progression period for 1st-2nd-3rd lines (times per month)^a^	Terminal careBeyond 3rd line period (times per month)^b^	Disease progression/One-off resource use at 3-month period (times)^c^
Medical consultations			
Medical oncologist consultation	0.73	1.6	3
Radiation oncologist consultation	0.083		
Oncology nurse visit	0.66	1.3	3
GP consultation	0.2	1	1
Psychology specialist consultation	0.08		
Surgeon consultation	0.08		
Hospital visits			
Inpatient stay (oncology/general ward)	0.06		2
Emergency department visit	0.083		
Day hospital visit	0.66	0.5	3
Home care			
Best supportive care physician/nurse visit		2	
Home aid (non-medical specialist) visit		2.3	
Examinations			
Whole-body CT	0.32		1
Brain MRI	0.033		
Brain CT-scan	0.016		
Chest radiograph	0.13		
PET-CT scan	0.033		
Bone scan	0.05		
Blood test (CBC, CMP)	1		3.71
Procedures			
Radiotherapy fraction	0.033		
Surgical intervention	0.033		

^a^For the pre progression period for 1st-2nd-3rd line, resource use is counted on monthly basis,

^b^For terminal care/beyond the 3rd line period, resource use is counted on monthly basis

^c^For the disease progression phase a 3-month period has been estimated for resource use based on the agreement of the advisory board participants

The most common resource use was the medical oncologist consultation (0.73 times per month), oncology nurse visit (0.66 times per month) and day hospital visit (0.66 times per month). Also, patients perform complete blood count lab test on a monthly basis. Patients at progression of disease perform 3 visits at hospital and 3 to oncologist. Patients beyond the third line usually use home aid visits (2.3 times per month). The cost of management during preprogression per month was estimated at €180.61, at progression €582.62 and beyond third line €72.58.

Table 4 presents the resource utilization during stable disease, at pre-progression, disease progression and beyond third line. Experts agreed that in the 2nd and 3rd line hospitalization and day hospital visits were increased. More specifically, hospitalization was 0.075 times in 2nd line and 0.105 times in the 3rd line and day hospital visits was 0.82 times and 1.15 times respectively. So, the cost of management at 2nd line per month was estimated at €188.21 and at 3rd line €203.81.

**Fig. 1:**
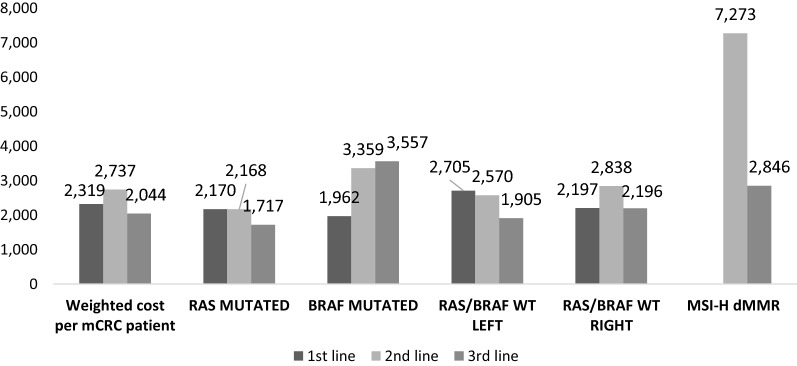
Monthly cost of mCRC patient per mutation and line

In Fig. 1 the monthly cost of mCRC patient per mutation and line is presented. At 1st and 2nd line, RAS mutated patients who receive combination of chemotherapy and Bevacizumab have the highest cost following by Regorafenib or Tas102 at 3rd line. Regarding BRAF mutated patients receiving chemotherapy and Bevacizumab at 1st line and those receiving immunotherapies as monotherapy and combination therapy of Encorafenib-Cetuximab at 2nd and 3rd line respectively have the highest cost of the respective category. The cost of 1st and 3rd line RAS/BRAF WT left mutated patient is attributed to combination of chemotherapy and Cetuximab, combination of chemotherapy and biological therapy or immunotherapies at 2nd line. The beyond third line treatment cost per patient regardless mutation was estimated at €1636.41 per month, which is mostly attributed to the cost of Regorafenib.

Finally, the weighted annual cost of mCRC patient at 1st line was estimated at €28,407, at 2nd line €33,568 and at 3rd line €25,550. In the annual cost were added the costs of 1 relapse (€582.62) at 1st line, 1.25 relapses (€728.27) at 2nd line and 1.75 relapses (€1019.58) at 3rd line.

## Discussion

Colorectal cancer is one major causes of cancer deaths, despite the advances in the management of patients with operable stage I-III cancers. Regarding metastatic CRC, the 5-year survival rate is between 13.8 and 14.7% for stage IV CRC patients [21]. Therefore, new treatments that could increase survival rate should be considered by health care decision makers as indispensable. Although the current analysis focuses on the cost of the disease, and mCRC seems to be a high-cost disease area, additional emphasis should be based on the survival benefits and improvement in patients’ quality of life that the new pharmaceutical advancements have brought in mCRC patients’ lives.

Τhe availability of modern targeted treatments enable patients with metastatic CRC to have their lives extended by months or even several years with improved quality. Over the last decade the median overall survival (OS) for patients with mCRC has increased from approximately 12 months, for those treated with a 5-fluorouracil (5-FU)-based chemotherapeutic regimens, to 18 months with the addition of irinotecan and oxaliplatin [22]. Additionally, biological targeted therapies such as cetuximab, panitumumab and bevacizumab in combination with chemotherapy, have increased the OS of mCRC to more than 24 months [23–25], although lead to high costs for the health care systems.

The present study investigated and provided an overall view of the resources use and associated costs required to treat metastatic CRC patients in Greece. This is the first study to analytically map all costs associated with the management of the disease in the local health care setting.

According to our results, at 1st, 2nd and 3rd line treatment the majority of patients receive biological targeted therapies in combination with chemotherapy (74.8%, 58.6% and 56% respectively) regardless of the mutation status. Resource utilization of patient management is important in order to be able to identify the respective cost of disease. Concerning resource utilization during stable disease (pre progression), the most usual resource consumption seems to be at medical oncologist consultation, oncology nurse visit and day hospital visits. Moreover, patients perform 17.3 oncologist visits per year and the same number in hospital at progression, and 13 visits per year in oncologists and in hospital beyond third line. Approximately 8–10% is the distribution of cost of resource use in all lines and mutations. The cost of resource utilization beyond third line is high at progression of disease, almost triple compared to monthly resource use. Monthly costs per mCRC patient, incurred by the Greek National Services Organization (EOPYY), were estimated on average for all mutations, at €2319 for the 1st line, €2737 for the 2nd line and €2044 for the 3rd line. On an annual basis, the weighted cost of mCRC patient at 1st line was estimated at €27,340, at 2nd line €33,568 and at 3rd line €25,550. RAS/BRAF WT Left had the highest cost at first line corresponding to 17% difference versus the average weighted cost, at second line MSI-H dMMR cost was more than two times higher (166%) compared to the average weighted cost while at third line BRAF Mutation cost was 74% higher than the average weighted cost.

At 1st line, the majority of patients, regardless the mutation status, receive bevacizumab in combination with chemotherapy (RAS mutated by 84%, BRAF mutated by 60%, RAS/BRAF WT LEFT by 30% and RAS/BRAF WT RIGHT by 45%). The only exception is RAS/BRAF WT LEFT where panitumumab based therapies are accounted for 40%. As a result, the main cost drive for the 1st line is bevacizumab-based therapies except RAS/BRAF WT LEFT were panitumumab-based therapies have also a significant impact.

At 2nd line, each mutation has different drivers towards cost. In patients *RAS* mutated tumors bevacizumab-based therapies, remain the key cost driver with 55%, while immunotherapies represent 5% of the prescribed therapies. In *BRAF* mutation, bevacizumab based therapies are accounted for 25%, combination of encorafenib plus cetuximab for 25% and immunotherapies for 10%. Regarding *RAS/BRAF* WT LEFT bevacizumab based therapies account for 68% and immunotherapies for only 2%. In *RAS/BRAF* WT RIGHT bevacizumab based therapies account for 30%, panitumab based therapy for 25% and immunotherapies for 10%. Finally, in MSI-H dMMR 100% of prescriptions are immunotherapies.

Based on the above, in 1st line, a factor that increases cost of *RAS/BRAF* WT LEFT above the weighted average, by almost 17%, is the increased percentage of panitumumab. In 2nd line *BRAF* mutation and *RAS/BRAF* WT RIGHT mutation are the categories that are above the weighted average, by almost 23% and 4% respectively. For BRAF mutation, the key driver of this excess can be assumed to be the increased rate of immunotherapies usage compared to other mutations and the introduction of encorafenib in combination with cetuximab to patients. For *RAS/BRAF* WT RIGHT mutation, immunotherapies and panitumumab are the key cost drivers.

It is important to note that comparison of mutation and line with the corresponding weighted average cannot contribute to a holistic approach regarding patients’ needs per mutation and line. As mCRC is a heterogeneous disease each mutation presents diverse clinical challenges and physicians have different treatment options available in order to improve patients’ OS and PFS [21]. For example, BRAF^V600E^ accounts for more than 90% of BRAF mutations in CRC [26] and a new targeted therapy, currently approved for 2nd line, for this mutation has been recently introduced in the Greek market (encorafenib plus cetuximab). The clinical data have demonstrated a statistically significant increase of the median overall survival (OS: 9.30 vs. 5.88 months), an improvement of overall response rate compared with chemotherapy plus cetuximab (19.5% vs 1.8%), and a longer increase in the progression-free survival (median PFS: 4.3 vs 1.5 months) in the BEACON study [27, 28].

The annual direct cost of mCRC patient according to our results on average for all lines is approximately 29,175 euros (2319 1st line, 2737 2nd line, 2044 3rd line on a monthly basis) which is slightly higher compared to other European studies. Specifically, a study conducted in Netherlands reported that the cost of metastatic cancer was estimated at €24,600 [29] and a Spanish study at €22,403 annually [30]. This difference may be attributed to the innovative biological therapies that were introduced and established as standard of care after the studies’ publication. However, United States cost for mCRC accounted at $41,562 and this higher cost is due to higher unit costs than in Europe as well as a clinical practice with greater use of resources [31].

The current study includes only direct medical costs, which reflect partly the total economic burden of mCRC. Indirect costs constitute another significant component of the societal burden of the disease, which accounts for a third of the total cost of the disease according to a study by Cole et al. [15]. In addition, CRC had the second-highest productivity losses due to mortality of all cancers in the EU, reaching €3.77 billion annually in 2009 (9% of the €42.6 billion in productivity losses from of all cancers [14]. In 2015, an estimation of costs due to loss of productivity because of either mortality or morbidity of CRC in Europe ranged from €1341 in Germany to €397 in Italy [15]. Moreover, the costs of informal care also represent an important cost component of the economic burden of CRC. These costs were high for CRC patients in the EU, reaching an annual €2.84 billion in 2009 (12% of the €23.2 billion total informal care provided in the EU) [14]. Additionally, the OHE estimated the annual costs of informal care for CRC per country in 2015 from €294 million in the UK to €871 million in Germany [15].

Although the treatment options for CRC have developed considerably over recent years, prevention of the disease is clearly the preferred option for individuals as well as all of society. In 2012, the European Union drew up guidelines on CRC screening and diagnosis, recommending the use of national screening programs based on fecal immunochemical test (FIT), endoscopic tests consist of flexible sigmoidoscopy or colonoscopy [32]. Since survival from effective treatment for early CRC exceeds 90% [33], it is feasible that formal population screening can significantly reduce mortality of this disease.

### Study limitations

There are certain limitations in the current study. The current concept for the management of mCRC is that of the continuum of care, which consist in a treatment strategy throughout the whole disease course, with de-escalation of treatment, maintenance treatment, treatment’s temporary discontinuations and reuse/rechallenge of treatment used previously. For this reason, there is no clear distinction between treatment lines in several cases. The direct cost of mCRC patient was estimated on monthly and annual basis, without taking into consideration the survival rate of each treatment, which is an important parameter for the choice of medical decision makers and the anticipating reason for the choice of targeted therapies as well as immunotherapies. Moreover, similar disease management/resource consumption was considered for all mCRC patients independent of mutation. In addition, this study does not take into consideration patients’ quality of life and how this is improved by the new treatments versus chemotherapy or other similar therapeutic regimens. Still, the decision-making process in healthcare should be based on cost-effectiveness criteria, in order for the policy maker to be able to balance both costs and outcomes and conclude in the adoption or not of new cost-effective treatments in local treatment algorithms.

However, despite the limitations, this study can serve as a basis for future economic evaluation studies in the management mCRC in Greece. The outcomes of this research can help the reader understand the local management and associated costs of mCRC and provide input for health care decision making in Greece, especially in the current period, where Health Technology Assessment (HTA) and negotiation committee are active in an effort to control the pharmaceutical expenditure.

## Conclusion

The cost of mCRC patient among lines was estimated to be similar regardless mutation. The highest cost of treatments is attributed to immunotherapy and targeted therapies which have improved significant patients’ clinical outcomes. Thus, treatment reimbursement decisions for patients with metastatic CRC should be based on the benefit that the treatment provides to the individual and their family, as well as to society. This cost analysis is a baseline study that will provide a useful source of information for informing decision-makers in terms of the allocation of resources and implementation of specific treatments and future studies on cost-effectiveness of different therapeutic innovations in Greece. The results of the study are also emphasizing the importance of screening protocols in asymptomatic population, which may lower the prevalence of the disease (removal of precancerous lesions/polys) and shifts diagnosis in early stages.

## Supplementary Information

**Additional file 1.** Questionnaire.

## Data Availability

Not applicable.
