# Supplementary material for: Treatment pathways and associated costs of metastatic colorectal cancer in Greece
Source: Cost Eff Resour Alloc. 2022 Feb 14;20:7. doi: 10.1186/s12962-022-00339-2 (PMC8842737; doi:10.1186/s12962-022-00339-2)
Supplement: Supplementary file 1 — Additional file 1. Questionnaire. [file 12962_2022_339_MOESM1_ESM.docx]

Additional file 1

QUESTIONNAIRE

Physician’s Information:

**Public Hospital:**  YES / NO (Please circle or underline or make bold)

**Region:** Please complete ………………………………………

**Number of patients with metastatic colorectal cancer on a yearly basis:** …….

**How many of those reported above are newly diagnosed per year:**……

Patient Epidemiology & Characteristics

**Question 1. Can you please provide reference for the incidence rate of CRC in Greece? Rate per 1000.**

Table 1: Incidence of CRC

|  | Incidence CRC Greece |
| --- | --- |
| Male |  |
| Female |  |
| Both |  |

**Question 2. Please provide the population parameters of mCRC patients**

Table 2: Population parameters

| Parameters | % of patients |
| --- | --- |
| % of metastatic CRC (mCRC) among CRC patients |  |
| % of BRAF mutation among mCRC patients |  |
| % of RAS mutation among mCRC patients |  |
| % of RAS/BRAF WT left mutation among mCRC patients |  |
| % of RAS/BRAF WT right mutation among mCRC patients |  |
| % of MSI-H dMMR mutation among mCRC patients |  |
| % of patients diagnosed and eligible for treatment |  |
| % of patients having a 2nd line of treatment |  |
| % of patients having a 3rd line of treatment |  |
| % of patients having a 4^th^ line or best supportive care |  |

**Question 3. Please complete the GR patient characteristics**

Table 3: Base-case cohort characteristics at baseline / GR patient characteristics

|  | GR PATIENT CHARACTERISTICS |
| --- | --- |
| Age (SD) |  |
| Height (SD) |  |
| Weight (SD) |  |
| BSA (SD) |  |
| Percentage males |  |

**Abbreviations**: BSA, body surface area; CSR, clinical study report; SD, standard deviation.

Source: BEACON CRC CSR

Treatment Strategies

#### AVAILABLE TREATMENTS AT FIRST LINE

**Question 4. Please let us know which option you use at 1st line and at which % (Your choices need to sum up to 100%)**

**Table 4: Treatment Options At 1st Line**

| 1^st^ line | RAS MUTATED | BRAF MUTATED | RAS/BRAF WT LEFT | RAS/BRAF WT RIGHT |
| --- | --- | --- | --- | --- |
| Folfiri |  |  |  |  |
| Folfiri + Cetuximab |  |  |  |  |
| Folfiri + Panitumumab |  |  |  |  |
| Folfiri + Bevacizumab |  |  |  |  |
| Folfox |  |  |  |  |
| Folfox + Cetuximab |  |  |  |  |
| Folfox + Panitumumab |  |  |  |  |
| Folfox + Bevacizumab |  |  |  |  |
| Folfoxiri |  |  |  |  |
| Folfoxiri + Cetuximab |  |  |  |  |
| Folfoxiri + Panitumumab |  |  |  |  |
| Folfoxiri + Bevacizumab |  |  |  |  |
| Capecitabine and Oxaliplatin (CapOx) |  |  |  |  |
| CapOx + Bevacizumab |  |  |  |  |
| CapOx + Cetuximab |  |  |  |  |
| CapOx + Panitumumab |  |  |  |  |
| Capecitabine + Bevacizumab |  |  |  |  |
| Capecitabine |  |  |  |  |
| TOTAL | **100** | **100** | **100** | **100** |

#### AVAILABLE TREATMENTS AT SECOND LINE

**Question 5. Please let us know which option you use at 2nd line and at which % (Your choices need to sum up to 100%)**

**Table 5: Treatment Options At 2nd Line**

|  | RAS MUTATED | BRAF MUTATED | RAS/BRAF WT LEFT | RAS/BRAF WT RIGHT | MSI-H  dMMR |
| --- | --- | --- | --- | --- | --- |
| Folfiri |  |  |  |  |  |
| Folfiri + Cetuximab |  |  |  |  |  |
| Folfiri + Panitumumab |  |  |  |  |  |
| Folfiri + Bevacizumab |  |  |  |  |  |
| Folfiri + Aflibercept |  |  |  |  |  |
| Folfox |  |  |  |  |  |
| Folfox + Cetuximab |  |  |  |  |  |
| Folfox + Panitumumab |  |  |  |  |  |
| Folfox + Bevacizumab |  |  |  |  |  |
| Folfoxiri |  |  |  |  |  |
| Folfoxiri + Cetuximab |  |  |  |  |  |
| Folfoxiri + Panitumumab |  |  |  |  |  |
| Folfoxiri + Bevacizumab |  |  |  |  |  |
| Capecitabine and Oxaliplatin (CapOx) |  |  |  |  |  |
| CapOx + Bevacizumab |  |  |  |  |  |
| CapOx + Cetuximab |  |  |  |  |  |
| CapOx + Panitumumab |  |  |  |  |  |
| Capecitabine + Bevacizumab |  |  |  |  |  |
| Capecitabine |  |  |  |  |  |
| Immunotherapies |  |  |  |  |  |
| Encorafenib-Cetuximab |  |  |  |  |  |
| Total | **100** | **100** | **100** | **100** |  |

#### AVAILABLE TREATMENTS AT THIRD LINE

**Question 6. Please let us know which option you use at 3rd line and at which % (Your choices need to sum up to 100%)**

**Table 6: Treatment Options At 3rd Line**

|  | RAS MUTATED | BRAF MUTATED | RAS/BRAF WT LEFT | RAS/BRAF WT RIGHT | MSI-H  dMMR |
| --- | --- | --- | --- | --- | --- |
| Folfiri |  |  |  |  |  |
| Folfiri + Cetuximab |  |  |  |  |  |
| Folfiri + Panitumumab |  |  |  |  |  |
| Folfiri + Bevacizumab |  |  |  |  |  |
| Folfox |  |  |  |  |  |
| Folfox + Cetuximab |  |  |  |  |  |
| Folfox + Panitumumab |  |  |  |  |  |
| Folfox + Bevacizumab |  |  |  |  |  |
| Capecitabine + Oxaliplatin (CapOx) |  |  |  |  |  |
| CapOx + Bevacizumab |  |  |  |  |  |
| CapOx + Cetuximab |  |  |  |  |  |
| CapOx + Panitumumab |  |  |  |  |  |
| Capecitabine + Bevacizumab |  |  |  |  |  |
| Capecitabine |  |  |  |  |  |
| Mitomycin-C |  |  |  |  |  |
| Cetuximab |  |  |  |  |  |
| Panitumumab |  |  |  |  |  |
| Tas102 |  |  |  |  |  |
| Regorafenib |  |  |  |  |  |
| Immunotherapies |  |  |  |  |  |
| Encorafenib + Cetuximab |  | 50 |  |  |  |
| Total | **100** | **100** | **100** | **100** | **100** |

#### AVAILABLE TREATMENTS AT TERMINAL CARE/ BEYOND 3rd LINE PERIOD

**Question 7. Please let us know which option you use at 4th line or BSC and at which % (Your choices need to sum up to 100%)**

**Table 7: Treatment Options At 4th Line or BSC**

|  | All patients |
| --- | --- |
| Folfiri |  |
| Folfiri + Cetuximab |  |
| Folfiri + Panitumumab |  |
| Folfiri + Bevacizumab |  |
| Folfox |  |
| Folfox + Cetuximab |  |
| Folfox + Panitumumab |  |
| Folfox + Bevacizumab |  |
| Capecitabine and Oxaliplatin (CapOX) |  |
| CapOX + Bevacizumab |  |
| CapOX + Cetuximab |  |
| CapOX + Panitumumab |  |
| Capecitabine + Bevacizumab |  |
| Capecitabine |  |
| Mitomycin-C |  |
| Cetuximab |  |
| Panitumumab |  |
| Tas102 |  |
| Regorafenib |  |
| Immunotherapies |  |
| TOTAL | **100** |

**Question 8. Please kindly report the % of your patients with metastatic CRC you perform surgery (%) and radiation therapy (%) irrespective of line of treatment**

**Table 8: Metastatic CRC patients having surgery and/or radiation irrespective of treatment line (%)**

|  | % of each category | |
| --- | --- | --- |
| Surgical intervention | | ……………………% |
| Radiotherapy fraction | | ……………………% |

### Resource Utilization During Stable Disease (pre progression, disease progression & terminal care )

**Question 9. Please let us know which resources are used in Greece during stable pre progression,** **disease progression and terminal care phase per month.**

Table 9: Routine management during stable disease pre-/post-progression

|  | Pre progression period for 1^st^-2^nd^-3^rd^ lines  (times per month) | Terminal care/ Beyond 3^rd^ line period (times per month) | Disease progression/ One-off resource use at 3-month period  (times) |
| --- | --- | --- | --- |
| Medical consultations |  |  |  |
| Medical oncologist consultation |  |  |  |
| Radiation oncologist consultation |  |  |  |
| Oncology nurse visit |  |  |  |
| GP consultation |  |  |  |
| Psychology specialist consultation |  |  |  |
| Surgeon consultation |  |  |  |
| Hospital visits |  |  |  |
| Inpatient stay (oncology/general ward) |  |  |  |
| Emergency department visit |  |  |  |
| Day hospital visit |  |  |  |
| Home care |  |  |  |
| Best supportive care physician/nurse visit |  |  |  |
| Home aid (non-medical specialist) visit |  |  |  |
| Examinations |  |  |  |
| Whole-body CT |  |  |  |
| Brain MRI |  |  |  |
| Brain CT-scan |  |  |  |
| Chest radiograph |  |  |  |
| PET-CT scan |  |  |  |
| Bone scan |  |  |  |
| Blood test (CBC, CMP) |  |  |  |

**THANK YOU VERY MUCH FOR YOUR ACTIVE PARTICIPATION IN OUR ADVISORY BOARD**
